# Prognosis according to clinical and pathologic lymph node status in breast cancer patients who underwent sentinel lymph node biopsy alone after neoadjuvant therapy

**DOI:** 10.1371/journal.pone.0251597

**Published:** 2021-05-18

**Authors:** Sae Byul Lee, Hakyoung Kim, Jisun Kim, Il Yong Chung, Hee Jeong Kim, Beom Seok Ko, Jong Won Lee, Sei Hyun Ahn, Byung Ho Son

**Affiliations:** Division of Breast Surgery, Department of Surgery, University of Ulsan College of Medicine, Asan Medical Center, Seoul, Korea; Fondazione Istituto G.Giglio di Cefalu, ITALY

## Abstract

This study aimed to evaluate the prognosis of breast cancer patients who received neoadjuvant chemotherapy and underwent sentinel lymph node biopsy (SLNB) alone as axillary surgery regardless of their clinical and pathological lymph node status. We reviewed the records of 1,795 patients from Asan Medical Center who were diagnosed with stage I–III breast cancer and received neoadjuvant chemotherapy during 2003–2014. We selected 760 patients who underwent SLNB alone as axillary surgery and divided these patients into four groups according to their clinical lymph node (cN) and pathological lymph node (pN) status: cN(-)pN(-) (n = 377), cN(-)pN(+) (n = 33), cN(+)pN(-) (n = 242), and cN(+)pN(+) (n = 108). We then compared axillary lymph node recurrence, locoregional recurrence (LRR), distant metastasis-free survival (DMFS), and overall survival (OS) among the four groups using Kaplan–Meier analysis. We compared prognosis between the cN(-)pN(-) and cN(+)pN(-) groups to determine whether SLNB alone is an adequate treatment modality even in patients with cN positive pathology before neoadjuvant therapy but SLNB-negative pathology after NAC. The 5-year axillary recurrence rates in the cN(-)pN(-) and cN(+)pN(-) groups were 1.4% and 2.9%, respectively, and there was no significant difference between the two groups (*p* = 0.152). The axillary recurrence and LRR rates were significantly different among the four groups, with the pN-negative groups (cN[–]pN[–], cN[+]pN[–]) showing lower recurrence rates. DMFS and OS were also significantly different among the four groups, with the cN negative groups (cN[–]pN[–], cN[–]pN[+]) showing improved survival rates. Our study findings suggest that SLNB alone was associated with lower LRR rates even in patients with cN positive pathology before neoadjuvant therapy but cN negative pathology after SLNB. Moreover, recurrence and survival rates differ significantly according to clinical and pathological lymph node status.

## Introduction

There has been an increase in the frequency of use of neoadjuvant chemotherapy (NAC) to minimize the surgical resection range in breast cancer patients who can be treated with surgery [1, 2]. Use of NAC can aid in the reduction of the size of the tumor; hence, breast-conserving surgery can be performed in patients who initially required total mastectomy. Moreover, NAC effectively lowers the stage of axillary lymph nodes (ALNs) [3]. During surgery, the occurrence of complications caused by axillary lymph node dissection (ALND) can be possibly prevented by identifying metastasized nodes through sentinel lymph node biopsy (SLNB), which is less invasive than ALND [4]. Several clinical studies have confirmed the accuracy of SLNB, and SLNB has become the standard surgical procedure to determine the axillary stage in primary breast cancer patients who have a clinically LN negative status [5, 6].

Several studies have been conducted to determine whether SLNB can accurately predict ALN metastasis after NAC in breast cancer patients. According to a recent study, the identification rate of ALN metastasis through SLNB in patients with NAC was 89.6%, and its false-negative rate (FNR) was 14.2% [7]. Since this FNR was higher than that in primary surgery patients, the oncological safety of SLNB-based surgery for these patients remains controversial.

Furthermore, pathological nodal response is an important component in determining the overall pathological response and nodal status in patients after NAC, which are closely associated with the patient’s prognosis. Therefore, accurately determining the nodal stage in a patient who has completed NAC is an important factor for evaluating the prognosis of the patient and for decision-making concerning adjuvant therapy [3]. In some studies, the nodal stage after NAC reflected the prognosis more accurately than the clinical axillary nodal stage before NAC [8]. However, only few long-term follow-up studies have been conducted in breast cancer patients after NAC.

Therefore, this study aimed to evaluate the prognosis of breast cancer patients who received NAC and underwent SLNB alone as axillary surgery regardless of their clinical and pathological LN status.

## Materials and methods

### Patients and clinical data

**Fig 1:**
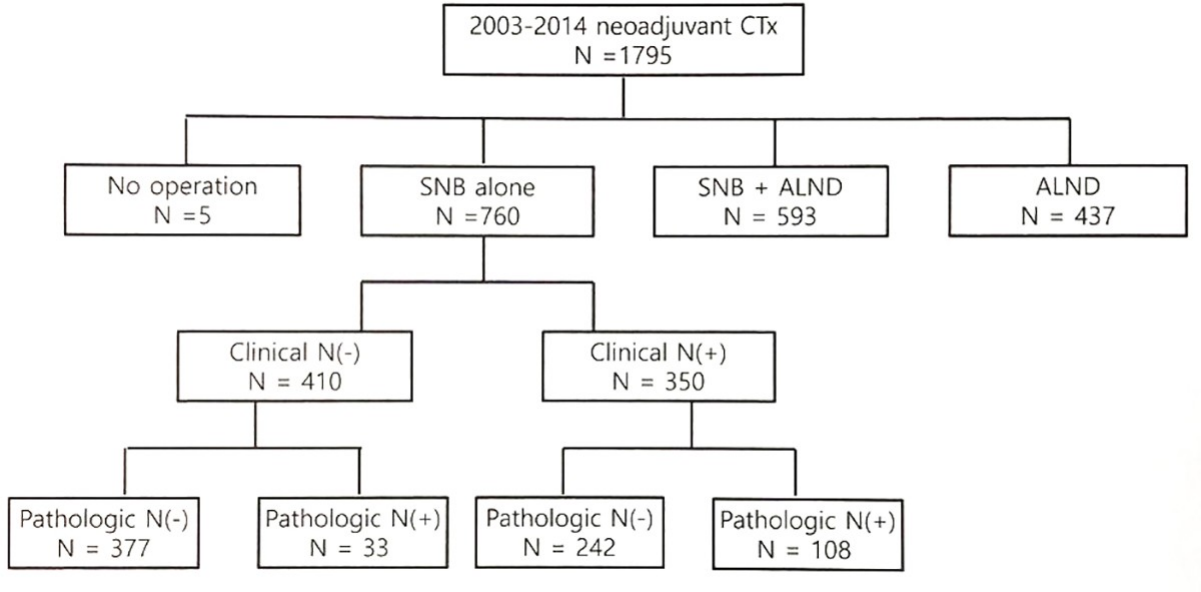
Flow diagram of the patient selection process. CTx chemotherapy, SLNB sentinel lymph node biopsy, ALND axillary lymph node dissection.

We reviewed the records of 1,795 patients from Asan Medical Center who were diagnosed with stage I–III breast cancer and received NAC during 2003–2014. Patients who had distant metastasis at the initial workup were excluded; 5 patients were excluded because they did not undergo surgery. A total of 593 patients who underwent ALND after SLNB and 437 patients who underwent ALND alone were also excluded. Finally, we conducted this study on 760 patients who underwent SLNB alone. In these patients, only SLNB was performed because the surgeon determined that no additional ALND was needed if the patient showed localized LN metastasis before chemotherapy and showed a good response to chemotherapy. The patients were divided into four groups according to their clinical LN status (cN) before NAC and pathological LN status (pN) after NAC: cN(-)pN(-) (n = 377), cN(-)pN(+) (n = 33), cN(+)pN(-) (n = 242), and cN(+)pN(+) (n = 108) (Fig 1). Information of all patient and tumor characteristics was retrieved from our prospectively collected database. The information collected in the database included data on patients’ age, clinical manifestations, clinical and pathological findings, surgical methods, types of adjuvant treatment modalities, type of recurrence, and follow-up period. This study was approved by the Institutional Review Board of Asan Medical Center, Seoul, South Korea (approval no. 20171341). The need for informed consent was waived because the study analyzed retrospective clinical data.

In the SLNB procedure, a colloidal radioisotope was administered on the day of surgery, some patients used lymphoscintigraphy and the sentinel LNs (SLNs) and non-sentinel LNs were excised. Moreover, additional sampling of LNs was permitted based on the surgeon’s decision.

NAC was administered using standard chemotherapy regimens for 3–6 months according to local guidelines or in accordance with ongoing study protocols. We considered patients to have positive axillary lymph nodes when metastasis to the node was demonstrated by core needle biopsy or fine needle aspiration biopsy. Pre-treatment (cTNM) and Post-treatment classification (ypTNM) was based on the definitions mentioned in the 7th edition of the American Joint Committee on Cancer staging system. cTNM was evaluated before the onset of NAC, and ypTNM was evaluated according to postoperative pathologic findings. Clinical nodal evaluation was performed using fine needle aspiration biopsy or core needle biopsy or considering imaging. The presence of isolated tumor cells (ypN0 [i]) was not considered as nodal metastasis.

### Statistical analyses

Data analyses were performed using the Statistical Package for the Social Sciences (SPSS), version 20.0 (SPSS Inc., USA). The chi-square test and t-test were used to determine the trends in each parameter. Axillary recurrence-free survival and locoregional recurrence-free survival were defined as the time from the date of the initial surgery to the date of the first appearance of an initial axillary recurrence and recurrence at the site of the primary tumor or locoregional lymph nodes, respectively.

Distant metastasis-free survival (DMFS) was defined as the time from the date of the initial surgery to the date of the first appearance of an initial metastasis. Overall survival (OS) was defined as the time from the initial surgery to the time of death. Survival curves were generated using the Kaplan–Meier method, and significant differences in survival according to the selected variables were verified using the log-rank test. All reported *p* values were two-sided, and a *p* value of <0.05 was considered statistically significant.

## Results

### Baseline characteristics

Data of the patients’ characteristics are presented in Table 1. The age at the initial operation in the entire study cohort (n = 760) was 45.1 years. Considering the clinical T stage before surgery, T2 was the most common (558 patients, 71.6%), followed by T3 (137 patients, 18.0%) and T1 (59 patients, 7.8%). Moreover, regarding clinical N stage, N0 was the most common (410 patients, 53.9%), followed by N1 (253 patients, 33.3%) and N3 (72 patients, 9.5%). The mean number of excised SLNs was 4.9. The distribution of pathological T stage after surgery was as follows: T1 (350 patients, 46.1%), T2 (182 patients, 23.9%), and T0 (154 patients, 20.3%). Additionally, 605 patients (79.6%) received radiotherapy, and 415 patients (54.6%) received hormonal therapy.

**Table 1:** Patient characteristics.

Factors	Total
	(N = 760)
	N (%)
Age at diagnosis (years)	
<35	114 (15.0)
35–50	419 (55.1)
>50	227 (29.9)
cT stage	
T1	59 (7.8)
T2	558 (73.4)
T3	137 (18.0)
T4	6 (0.8)
cN stage	
N0	410 (53.9)
N1	253 (33.3)
N2	25 (3.3)
N3	72 (9.5)
cStage	
I	22 (2.9)
II	596 (78.4)
III	142 (18.7)
Operation method	
BCS	509 (67.0)
Mastectomy	251 (33.0)
pT stage	
T0	154 (20.3)
Tis	54 (7.1)
T1	350 (46.1)
T2	182 (23.9)
T3	19 (2.5)
T4	1 (0.1)
Pathologic nodal status	
Negative	615 (80.9)
Positive	145 (19.1)
Number of excised LNs (Mean ± SD)	4.9 ± 2.6
CR status	
No	617 (81.2)
Yes	143 (18.8)
Histologic Grade	
G1	18 (3.2)
G2	293 (52.3)
G3	249 (44.5)
Unknown	200
Nuclear Grade	
G1	16 (2.7)
G2	305 (51.7)
G3	269 (45.6)
Unknown	170
Lymphovascular invasion	
No	478 (78.9)
Yes	128 (21.1)
Unknown	154
Ki-67	
<20	416 (68.6)
≥20	190 (31.4)
Unknown	154
ER	
Negative	273 (45.3)
Positive	329 (54.7)
Unknown	158
PgR	
Negative	417 (69.3)
Positive	185 (30.7)
Unknown	158
HER2 (IHC)	
Negative	428 (71.0)
Positive	175 (29.0)
Unknown	157
Chemotherapy regimen	
Anthracyclin based	323 (42.5)
Taxane-based	422 (55.5)
Others	15 (2.0)
Radiotherapy	
No	155 (20.4)
Yes	605 (79.6)
Hormonal therapy	
No	345 (45.4)
Yes	415 (54.6)

BCS = breast-conserving surgery; CR = complete response; ER = estrogen receptor; PgR = progesterone receptor; HER2 = human epidermal growth factor receptor 2; IHC = immunohistochemistry; LN, lymph node

The 760 patients who underwent SLNB alone after NAC were divided into 4 groups according to the cN before NAC and pN after NAC: cN(-)pN(-) (n = 377), cN(-)pN(+) (n = 33), cN(+)pN(-) (n = 242), and cN(+)pN(+) (n = 108) (Fig 1). Table 2 shows a comparison of patient characteristics according to lymph node status.

**Table 2:** Comparison of patients’ characteristics according to their lymph node status.

Factors	cN(-)pN(-)	cN(-)pN(+)	cN(+)pN(-)	cN(+)pN(+)	*p* value
	(N = 377)	(N = 33)	(N = 242)	(N = 108)	
	N (%)	N (%)	N (%)	N (%)	
Age at diagnosis (years)					0.045
<35	57 (15.1)	4 (12.1)	27 (11.2)	26 (24.1)	
35–50	217 (57.6)	18 (54.5)	136 (56.2)	48 (44.4)	
>50	103 (27.3)	11 (33.4)	79 (32.6)	34 (31.5)	
cT stage					0.018
T1	17 (4.5)	2 (6.1)	31 (12.8)	9 (8.3)	
T2	294 (78.0)	25 (75.7)	163 (67.3)	76 (70.4)	
T3	65 (17.2)	6 (18.2)	44 (18.2)	22 (20.4)	
T4	1 (0.3)	0 (0)	4 (1.7)	1 (0.9)	
cN stage					<0.001
N0	377 (100)	33 (100)	0 (0)	0 (0)	
N1	0 (0)	0 (0)	175 (72.3)	78 (72.2)	
N2	0 (0)	0 (0)	19 (7.9)	6 (5.6)	
N3	0 (0)	0 (0)	48 (19.8)	24 (22.2)	
cStage					<0.001
I	19 (5.0)	2 (6.1)	0 (0)	1 (0.9)	
II	357 (94.7)	31 (93.9)	145 (59.9)	63 (58.3)	
III	1 (0.3)	0 (0)	97 (40.1)	44 (40.7)	
Operation method					0.015
BCS	263 (69.8)	16 (48.5)	167 (69.0)	63 (58.3)	
Mastectomy	114 (30.2)	17 (51.5)	75 (31.0)	45 (41.7)	
pT stage					<0.001
T0	55 (14.6)	0 (0)	92 (38.0)	7 (6.5)	
Tis	21 (5.6)	0 (0)	28 (11.6)	5 (4.6)	
T1	178 (47.2)	14 (42.4)	97 (40.1)	61 (56.5)	
T2	115 (30.5)	15 (45.5)	22 (9.1)	30 (27.8)	
T3	8 (2.1)	4 (12.1)	3 (1.2)	4 (3.7)	
T4	0 (0)	0 (0)	0 (0)	1 (0.9)	
CR status					<0.001
No	324 (85.9)	33 (100)	152 (62.8))	108 (200)	
Yes	53 (14.1)	0 (0)	90 (37.2)	0 (0)	
Histologic Grade					0.013
G1	12 (4.0)	0 (0)	4 (3.1)	2 (2.1)	
G2	141 (46.5)	26 (78.8)	69 (53.5)	57 (60.0)	
G3	150 (49.5)	7 (21.2)	56 (43.4)	36 (37.9)	
Unknown	74	0	113	13	
Nuclear Grade					0.010
G1	9 (2.9)	1 (3.0)	4 (2.8)	2 (2.0)	
G2	143 (45.4)	24 (72.8)	75 (52.4)	63 (63.7)	
G3	163 (51.7)	8 (24.2)	64 (33.8)	34 (34.3)	
Unknown	62	0	99	9	
Lymphovascular invasion					<0.001
No	272 (84.5)	18 (54.5)	125 (83.3)	63 (62.4)	
Yes	50 (15.5)	15 (45.5)	25 (16.7)	38 (37.6)	
Unknown	55	0	92	7	
ER					<0.001
Negative	158 (49.2)	11 (33.3)	76 (51.7)	28 (27.7)	
Positive	163 (50.8)	22 (67.7)	71 (48.3)	73 (72.3)	
Unknown	56	0	95	7	
PgR					0.002
Negative	224 (69.8)	19 (57.6)	116 (78.9)	58 (57.4)	
Positive	97 (30.2)	14 (42.4)	31 (21.1)	43 (42.6)	
Unknown	56	0	95	7	
HER2 (IHC)					0.016
Negative	232 (72.0)	24 (72.7)	91 (61.9)	81 (80.2)	
Positive	90 (28.0)	9 (27.3)	56 (38.1)	20 (19.8)	
Unknown	55	0	95	7	
Ki-67					0.323
<20	214 (66.5)	22 (66.7)	103 (68.7)	77 (76.2)	
≥20	108 (33.5)	11 (33.3)	47 (31.3)	24 (23.8)	
Unknown	55	0	92	7	
Radiotherapy					0.030
No	81 (21.5)	12 (36.4)	38 (15.7)	24 (22.2)	
Yes	296 (78.5)	21 (63.6)	204 (84.3)	84 (77.8)	
Hormonal therapy					<0.001
No	182 (48.3)	10 (30.3)	125 (51.7)	28 (25.9)	
Yes	195 (51.7)	23 (69.7)	117 (48.3)	80 (74.1)	

BCS = breast-conserving surgery; CR = complete response; ER = estrogen receptor; PgR = progesterone receptor; HER2 = human epidermal growth factor receptor-2; IHC = immunohistochemistry

### Survival outcomes

**Fig 2:**
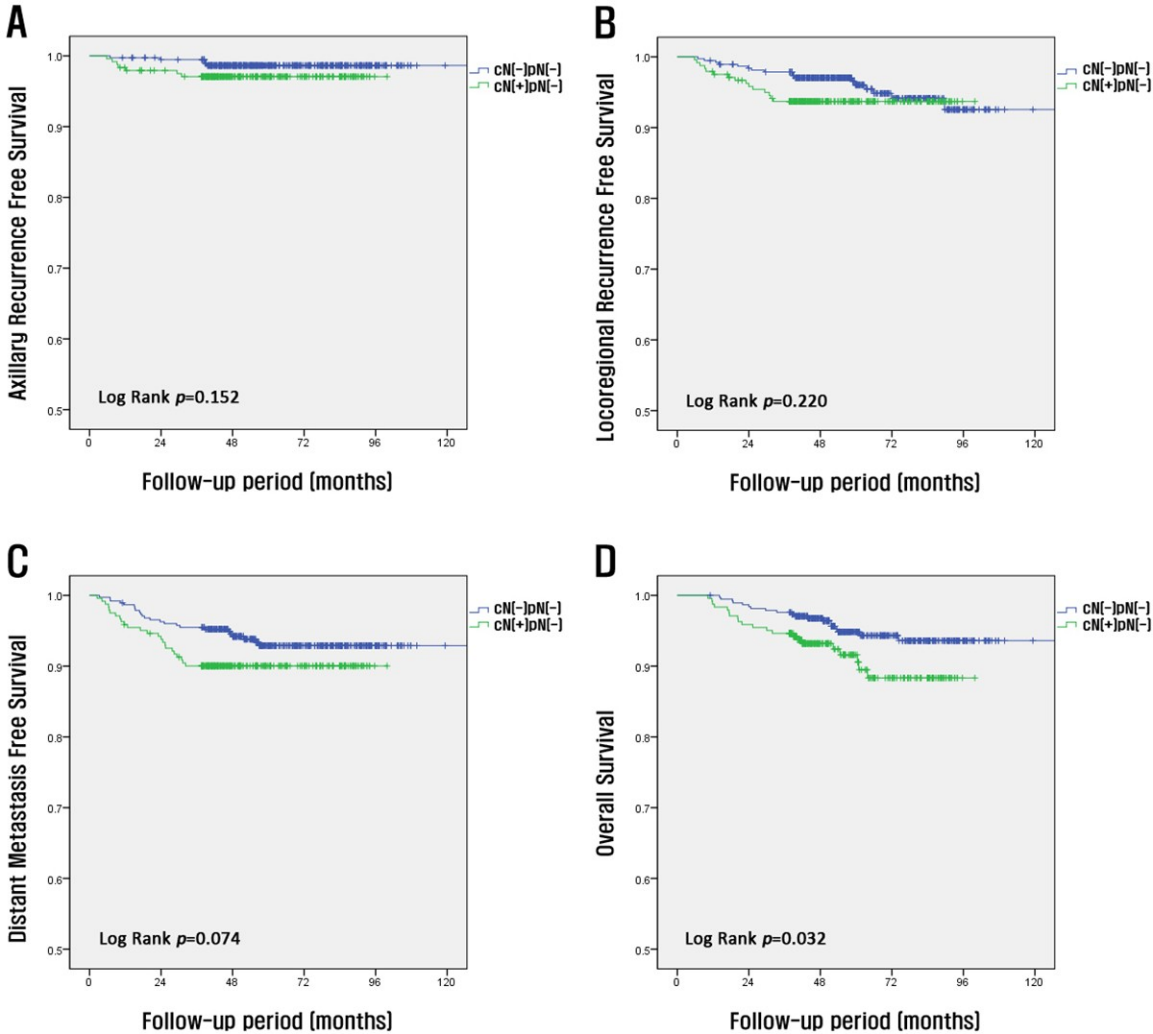
Kaplan–Meier curves according to clinical node status in patients with pathologically negative nodes (-). Axillary recurrence-free survival (A), locoregional recurrence-free survival (B), distant metastasis-free survival (C), and overall survival (D).

We compared the survival rates of patients with pathologically negative LNs detected postoperatively according to preoperative clinical node status (cN[–]pN[–] vs. cN[+]pN[–]). The 5-year axillary recurrence-free survival and 5-year locoregional recurrence-free survival rates of the corresponding groups were 98% and 97% (*p* = 0.152) and 96% and 95% (*p* = 0.220), respectively. Moreover, there were no significant differences between the two groups (Fig 2A and 2B). However, the 5-year DMFS rate (94% and 90%, respectively, *p* = 0.074) and 5-year OS rate (93% and 87%, respectively, *p* = 0.032) were significantly different between the two groups (Fig 2C and 2D).

**Fig 3:**
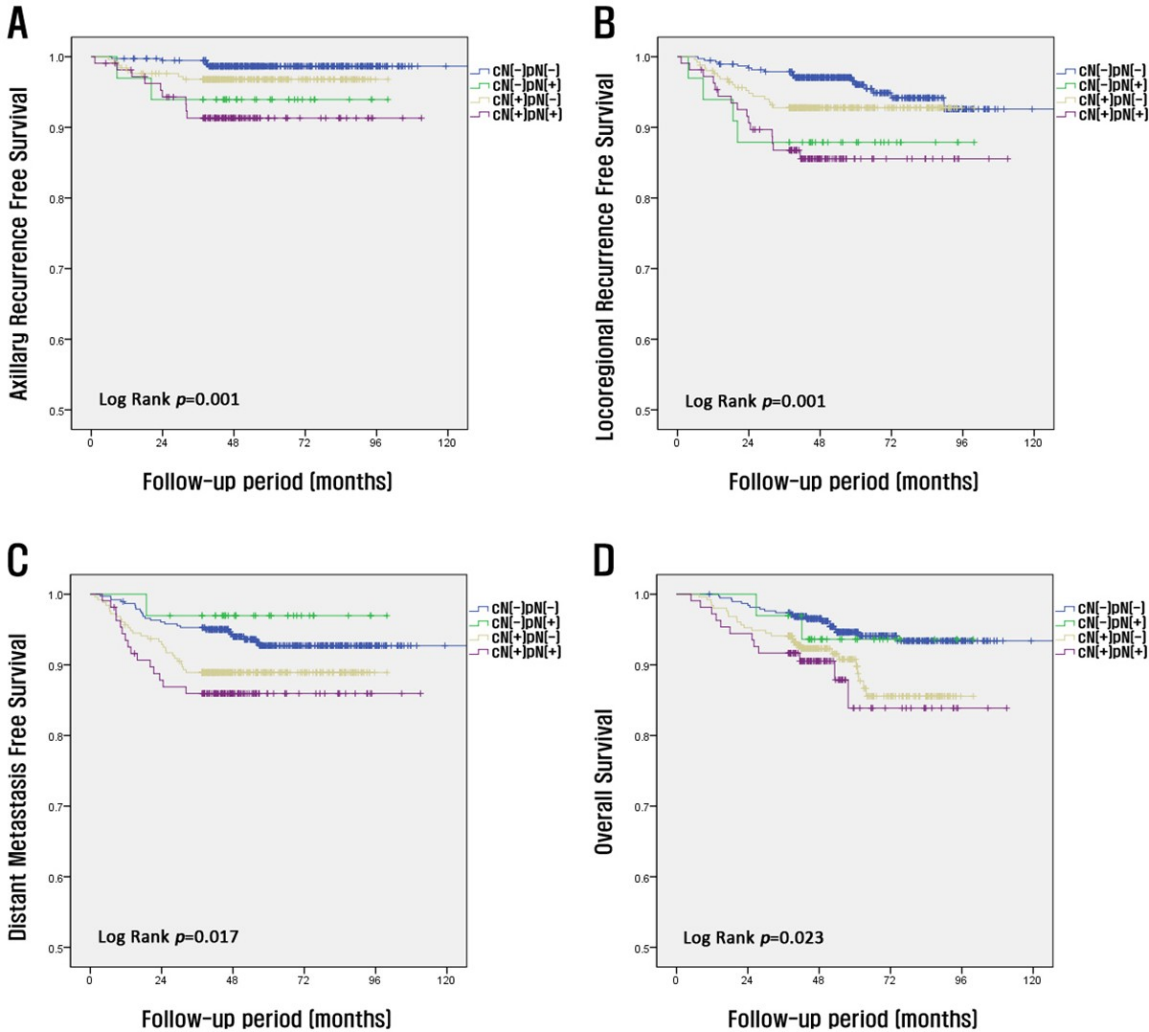
Kaplan–Meier curves according to patients’ clinical and pathological nodal status. Axillary recurrence-free survival (A), locoregional recurrence-free survival (B), distant metastasis-free survival (C), and overall survival (D).

The patient’s outcomes are shown in Fig 3; the four groups were divided based on clinical lymph LN and pathological LN status. The patterns of axillary recurrence-free survival and locoregional recurrence-free survival were significantly different from those of DMFS and OS. The axillary recurrence and locoregional recurrence rates were significantly different depending on the status of lymph node metastasis on postoperative pathology. The axillary recurrence-free survival and locoregional recurrence-free survival rates of the cN(-)pN(-) and cN(+)pN(-) groups were significantly higher than those of the cN(-)pN(+) and cN(+)pN(+) groups (*p* = 0.001 and *p* = 0.001, respectively). In contrast, the distant recurrence and OS rates were significantly different according to whether the LN were metastasized on clinical examination before NAC than on the pathological examination after surgery. The DMFS and OS rates of the cN(-)pN(-) and cN(-)pN(+) groups were significantly higher than those of the cN(+)pN(-) and cN(+)pN(+) groups (*p* = 0.017 and *p* = 0.023, respectively) (Fig 3).

The outcomes were then compared according to whether lymph node metastasis was noted on pathological examination after surgery (pN[–] vs. pN[+]) (S1 Fig). The 5-year axillary recurrence-free survival (98%, A) and 5-year locoregional recurrence-free survival (93%, B) rates in the pN(-) group were higher than those in the pN(+) group (92%, A; 85%, B), with a significant difference between the two groups (*p*<0.001 and *p*<0.001, respectively). In contrast, there was no significant difference between the two groups in the 5-year DMFS and 5-year OS rates (*p* = 0.185 and *p* = 0.120, respectively).

The outcomes showed different patterns according to whether or not clinical LN metastases was noted before surgery (cN[–] vs. cN[+]) (S2 Fig). There were no significant differences in the 5-year axillary recurrence-free survival (A) and 5-year locoregional recurrence-free survival (B) rates between the cN(-) and cN(+) groups (*p* = 0.075 and *p* = 0.072, respectively). However, the 5-year DMFS (95% and 89%, C, respectively) and 5-year OS (94% and 85%, D) rates were significantly different between the two groups (*p* = 0.007 and *p* = 0.004, respectively).

## Discussion

ALN metastasis is one of the important factors that determine the prognosis of breast cancer patients. The ALND can determine the ALN stage and affect locoregional control, but it can lead to serious complications such as lymphedema or nerve damage. As an alternative, SLNB, a procedure associated with minimal complications, has become the standard method for primary breast cancer management since its introduction [9].

However, the number of patients receiving NAC before surgery is increasing, and in recent years, owing to improvement in the effect of systemic chemotherapy, there has been an increase in the number of patients with a good response. Considering these changes, the approach to the treatment of ALNs must also be modified [3]. Several studies assessing the role and effectiveness of SLNB after NAC have suggested that SLNB performed after NAC may be a good option for minimizing surgical coverage [10–13]. In a meta-analysis of 21 studies, the average detection rate and FNR of SLNB were 91% and 12%, respectively [14]. Several studies and a meta-analysis of some studies showed the detection rate and FNR were similar between patients who underwent surgery after NAC and those who underwent upfront surgery [15, 16]. Moreover, the FNR was 7.3% in the SENTinel NeoAdjuvant trial and 9.1% in the American College of Surgeons Oncology Group Z1071 trial when three or more SLNs were retrieved [1]. These studies suggest that SLNB is acceptable for breast cancer patients after NAC.

Nodal recurrence is considered controversial when SLNB alone is performed in patients who test cN positive before NAC but test cN negative after NAC [17–19]. In our study, we compared the locoregional recurrence rate between patients with cN negative status both before and after NAC and patients with cN positive status before NAC but cN negative status after NAC; no significant difference in recurrence rate was noted. Therefore, we carefully conclude that the locoregional recurrence rate did not differ significantly between patients who underwent SLNB alone in whom the cN status changed from positive to negative after NAC and patients undergoing upfront surgery. The current National Comprehensive Cancer Network guidelines allow SLNB alone to be used for the staging of patients whose cN status has changed from positive to negative after NAC [20]. Moreover, studies including the National Surgical Adjuvant Breast and Bowel Project B-18 and B-27 have shown that the locoregional recurrence rate is low in patients with ypN0 status after NAC [21]. Kim et al. analyzed 386 patients with cytology-proven breast cancer before NAC and found no significant difference in axillary recurrence between the SLNB alone and ALND groups with a median follow-up duration of 19.5 months [22].

Currently, the association between axillary failure and distant metastasis or patient survival remains unclear. Only a few studies have assessed the long-term follow-up results of SLNB in such patients. Bilimoria et al. studied the differences in axillary recurrence and OS between patients undergoing SLNB alone and those undergoing SLNB and ALND among patients with pN1 disease after surgery [23]. The axillary recurrence and OS rates of patients who underwent SLNB alone were not significantly different from those of patients who underwent SLNB and ALND [24].

The present study compared the distant recurrence and OS rates between patients with cN negative status both before and after NAC who underwent SLNB alone and patients with cN positive status before NAC but cN negative status after NAC who underwent SLNB alone. The distant recurrence-free survival and OS rates were higher in patients with cN(-) status than in patients with cN(+) status. The analysis of the four groups divided based on clinical LN status and pathological LN status showed similar results. The axillary recurrence-free survival and locoregional recurrence-free survival rates of the cN(-)pN(-) and cN(+)pN(-) groups were significantly higher than those of the cN(-)pN(+) and cN(+)pN(+) groups (*p* = 0.001 and *p* = 0.001, respectively). The axillary recurrence and locoregional recurrence rates were significantly different depending on the status of lymph node metastasis on postoperative pathology. Furthermore, the distant metastasis-free survival and OS rates were different. The 5-year DMFS and OS rates were significantly different according to whether lymph node metastasis was observed on clinical examination before surgery or on pathological examination after surgery. Therefore, even if ALND is not required when N0 disease is confirmed after SLNB, further treatment should be considered because the distant recurrence rate and mortality rate are high in cN(+) patients.

Our results clearly demonstrate that pathological axillary nodal status has a major impact on the rates and patterns of axillary recurrence and locoregional recurrence. The results further suggest that absolute locoregional recurrence rates are low if a patient with pathologically negative ALNs achieves a pathological complete response. Some studies have reported that instead of evaluating the axillary stage before NAC, evaluating the stage after NAC shows better results for determining the patient’s residual disease severity and response to chemotherapy [3]. We believe these results will be of important help in determining additional chemotherapy, hormone therapy, radiation therapy, and targeted therapy for post-NAC patients. Also, it is helpful to use these results to organize complex treatments through clinical trials.

The limitations of this study were as follows: this study was a retrospective review of cases; hence, selection bias and surgeon bias could have affected the results. However, in the absence of a randomized controlled trial, we believe that our study is valuable because to the best of our knowledge, this study presents the long-term outcomes of patients who showed a clinically ALN positive status initially but a pathologic ALN negative status after NAC and underwent SLNB without ALND. Patients were classified according to their nodal response to NAC in our study. This method is more clinically practical and can help us reach more accurate conclusions than other methods. The possibility of false negative/positive results in both biopsy and pathological examination of lymph nodes is also a limitation. Nevertheless, this study was a single-center clinical trial that analyzed few cases compared to other studies.

In conclusion, our study shows that SLNB alone as associated with lower locoregional recurrence rates even in patients who showed a clinically LN positive status before NAC but a pathologically LN negative status after NAC. SLNB alone remains a useful method in breast cancer patients who show conversion from ALN positive pathology to ALN negative pathology after NAC. Moreover, there are significant differences in recurrence and survival rates according to clinical and pathological lymph node status.

## Supporting information

S1 Fig Kaplan–Meier curves according to pathological node status. Axillary recurrence-free survival (a), locoregional recurrence-free survival (b), distant metastasis-free survival (c), and overall survival (d).
(JPG)

S2 Fig Kaplan–Meier curves according to clinical node status. Axillary recurrence-free survival (a), locoregional recurrence-free survival (b), distant metastasis-free survival (c), and overall survival (d).
(JPG)

S1 File (XLSX)
