# Supplementary figures and images for: Prognosis according to clinical and pathologic lymph node status in breast cancer patients who underwent sentinel lymph node biopsy alone after neoadjuvant therapy
Source: PLoS One. 2021 May 18;16(5):e0251597. doi: 10.1371/journal.pone.0251597 (PMC8130919; doi:10.1371/journal.pone.0251597)

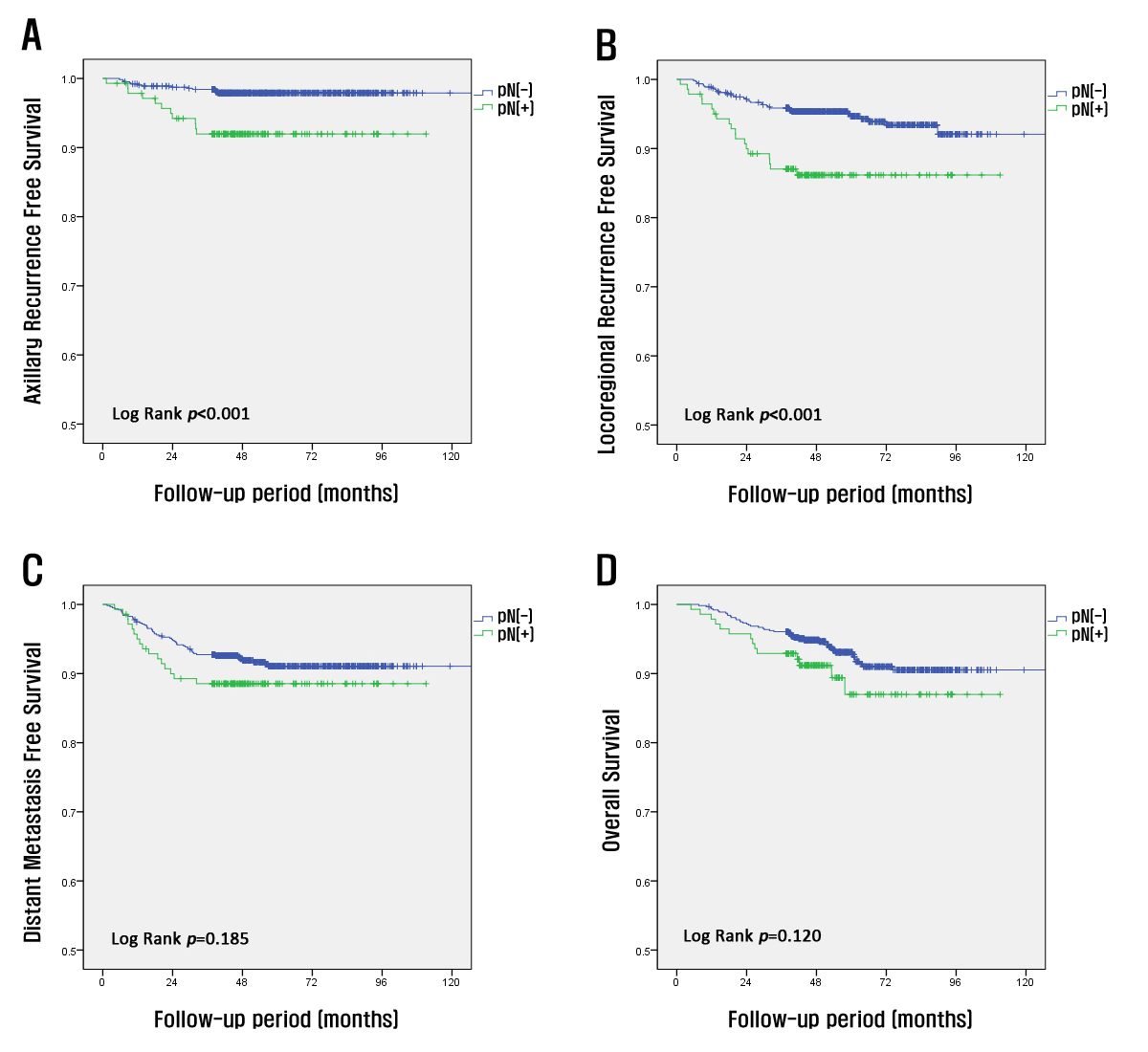

Supplement: S1 Fig — Axillary recurrence-free survival (a), locoregional recurrence-free survival (b), distant metastasis-free survival (c), and overall survival (d). (JPG) [file pone.0251597.s001.jpg]

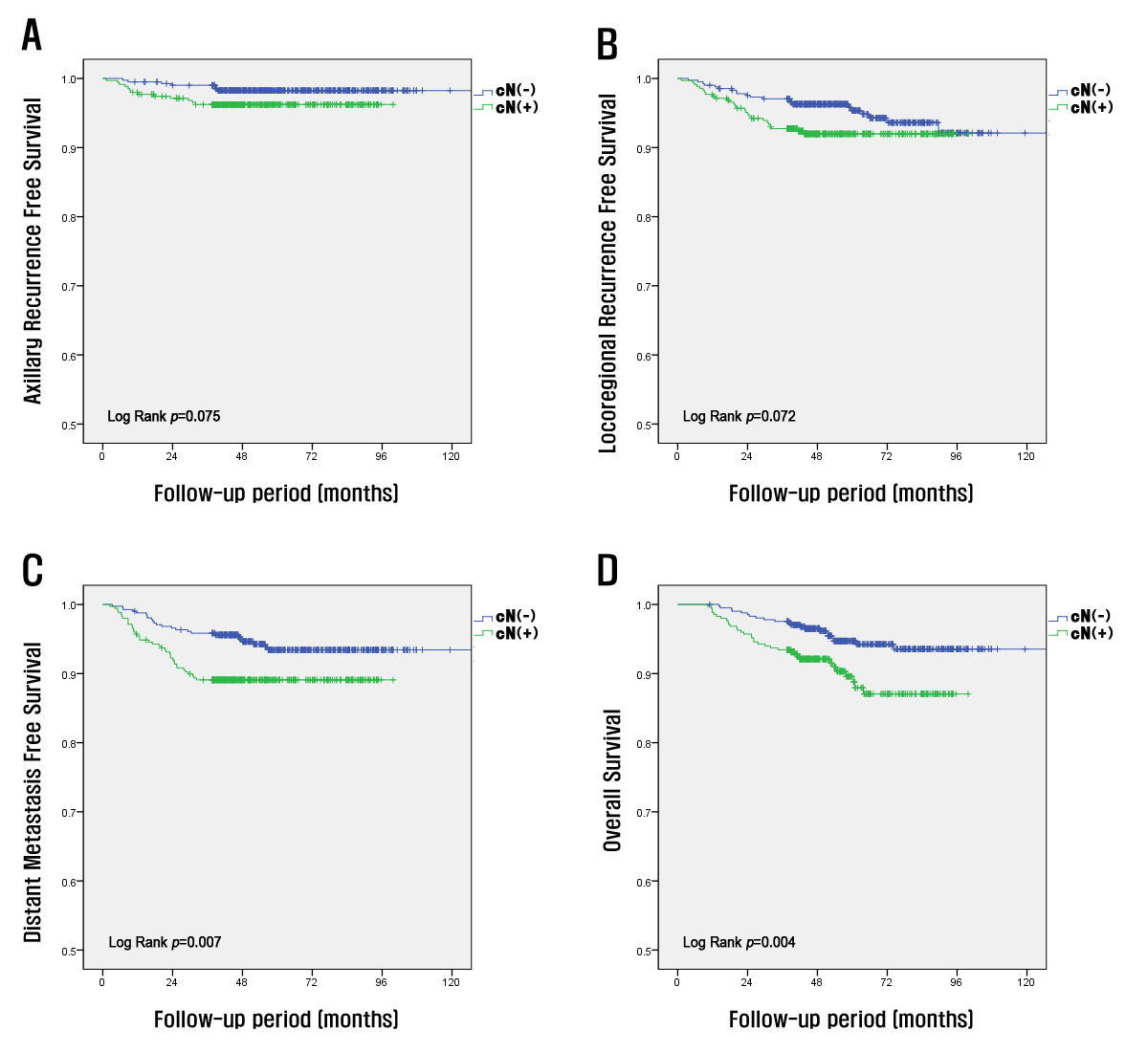

Supplement: S2 Fig — Axillary recurrence-free survival (a), locoregional recurrence-free survival (b), distant metastasis-free survival (c), and overall survival (d). (JPG) [file pone.0251597.s002.jpg]
